# Diagnostic Performance and Error Patterns of a Large Language Model and Neural Network in Periodontitis Classification: A Comparative Study

**DOI:** 10.3390/jcm15124837

**Published:** 2026-06-22

**Authors:** Agata Ossowska, Aida Kusiak, Albert Camlet, Dariusz Świetlik

**Affiliations:** 1 Department of Periodontology and Oral Mucosa Diseases, Medical University of Gdansk, 80-200 Gdansk, Poland; agata.ossowska@gumed.edu.pl (A.O.); aida.kusiak@gumed.edu.pl (A.K.); acamlet@gumed.edu.pl (A.C.); 2 Department of Biostatistics and Neural Networks, Medical University of Gdansk, 80-210 Gdansk, Poland

**Keywords:** periodontitis, artificial intelligence, neural networks, large language models, disease classification, diagnostics, diagnostic errors

## Abstract

**Background/Objectives**: Periodontitis is a highly prevalent chronic disease requiring accurate diagnosis for effective treatment planning. Artificial intelligence (AI) has emerged as a potential tool to support clinical decision-making. This study aimed to compare the diagnostic performance and classification error patterns of a large language model (LLM) and a neural network (NN) in periodontitis classification according to the current staging and grading system. **Methods**: This retrospective study included 110 patients with periodontal disease. Clinical and demographic variables (age, sex, smoking status, number of teeth, API, BOP, PPD, and CAL) were analyzed. Reference diagnoses were established by two experts. Cases were evaluated using an LLM and a neural network. Model performance was assessed using accuracy, confusion matrices, and Cohen’s kappa coefficient, along with error analysis. **Results**: The LLM achieved 62% accuracy for stage and 63% for grade classification (κ = 0.48). The neural network showed higher performance, with 85% accuracy for stage and 79% for grade (κ = 0.79 and κ = 0.67, respectively). The LLM more often underestimated disease severity, whereas the neural network tended to overestimate progression. Differences between models were statistically significant (*p* < 0.0001). **Conclusions**: In this dataset and classification task, the task-specific neural network demonstrated higher diagnostic performance than the evaluated large language model. However, the findings should be interpreted in light of the fundamentally different training paradigms and intended applications of these AI systems. Further research is required to optimize and validate AI-based approaches for clinical use.

## 1. Introduction

Periodontitis is a chronic inflammatory disease affecting the supporting tissues of the teeth and represents one of the most prevalent oral conditions worldwide [1,2]. It is characterized by the progressive destruction of the periodontal ligament and alveolar bone, ultimately leading to tooth loss and exerting a negative impact on systemic health [1,3]. Epidemiological evidence indicates that periodontal diseases rank among the most common chronic disorders globally and impose a substantial burden on healthcare systems [2,4].

Accurate diagnosis and classification of periodontitis are essential for appropriate treatment planning and prognosis assessment [5]. The current classification system, introduced in 2017, incorporates two key dimensions: staging, which reflects disease severity and complexity, and grading, which captures the rate of progression and the influence of risk factors [1,5]. Its application in clinical practice requires the integration of multiple clinical and radiographic parameters, including clinical attachment loss (CAL), probing depth (PD), radiographic bone loss, and patient-related risk factors such as smoking and diabetes [1,5]. However, the multifactorial nature of these assessments introduces potential variability, making diagnostic outcomes dependent on examiner experience and susceptible to inter-observer differences [6,7].

In recent years, artificial intelligence (AI) has emerged as a promising tool to support clinical decision-making in dentistry [8,9]. Contemporary discussions emphasize that AI systems should be regarded primarily as decision-support technologies intended to augment, rather than replace, clinician judgment, particularly in complex diagnostic environments requiring the integration of multiple variables [9,10]. In periodontology, artificial neural networks (ANNs) and deep learning models have demonstrated high performance in the analysis of both clinical and imaging data. Several studies have reported that such models can achieve diagnostic accuracy comparable to that of experienced clinicians, while offering advantages in reproducibility, scalability, and consistency across large datasets [10,11].

At the same time, the increasing incorporation of AI into clinical workflows has raised important concerns regarding transparency, accountability, and reliability in medical decision-making [12,13,14]. Many advanced AI systems, especially deep learning models, operate as “black boxes,” making it difficult for clinicians to fully understand the rationale underlying algorithmic outputs [14]. This limitation may affect trust, interpretability, and medico-legal responsibility in situations involving diagnostic errors or inappropriate treatment recommendations [13,14]. Consequently, the recent literature highlights the importance of evaluating not only overall diagnostic accuracy, but also the structure and clinical significance of AI-related errors in order to ensure safe and responsible implementation in healthcare settings [13,14,15].

Alongside advances in image-based AI, a new class of models based on natural language processing—large language models (LLMs)—has recently gained attention [12,16]. These models enable the processing of textual and structured clinical data and facilitate the integration of multiple parameters within complex decision-making workflows [12]. Preliminary evidence suggests that transformer-based models may be applicable to the classification of periodontitis using clinical data; however, their reliability, consistency, and interpretability in complex diagnostic settings remain insufficiently validated and require further investigation [16,17].

Despite the growing body of research on AI applications in periodontology, most studies focus on single-model approaches, predominantly relying on radiographic image analysis [9,18]. Direct comparisons between different AI paradigms, such as neural networks and large language models, remain limited [9,19]. Furthermore, beyond overall diagnostic accuracy, increasing attention is being directed toward the analysis of error patterns, as different types of misclassification may have distinct clinical implications and may influence clinician trust in AI-assisted decision support systems [19,20].

The aim of this study was to evaluate and compare the diagnostic performance and classification error patterns of a large language model and a neural network in the classification of periodontitis according to the current staging and grading system. This analysis provides deeper insight into the strengths and limitations of both approaches and facilitates the assessment of their potential utility in clinical practice. Unlike previous studies focusing primarily on overall accuracy, the present study additionally evaluated the qualitative structure of diagnostic errors, which may have important implications for the safe and responsible integration of AI-assisted diagnostic tools into periodontal care.

## 2. Materials and Methods

### 2.1. Study Design

The study was designed as a retrospective observational diagnostic accuracy study aimed at comparing the performance and diagnostic error patterns of two different types of artificial intelligence models: a large language model (LLM) and a neural network, in the classification of periodontal disease. The analysis was conducted using a set of standardized clinical cases describing diagnostic parameters of patients with periodontitis.

The primary objective of the study was to compare the classification accuracy and the characteristics of diagnostic errors made by both models when determining the stage and grade of periodontal disease according to the current periodontal disease classification.

### 2.2. Study Material

The study material consisted of a database of 110 patients of both sexes aged between 30 and 60 years. The study had a retrospective design and was based on the analysis of clinical records of patients treated for periodontal diseases. Patients were selected in 2025 at the Department of Periodontology and Oral Mucosa Diseases, Medical University of Gdańsk. Clinical data were obtained from patients’ medical records and anonymized prior to the analysis.

### 2.3. Clinical and Demographic Variables

Demographic data and clinical parameters used in the diagnosis of periodontal diseases were obtained from the patients’ medical records. The following variables were included in the analysis:

- Sex—a categorical variable indicating the patient’s sex.
- Age—the patient’s age in years at the time of the clinical examination.
- Smoking status—information on cigarette smoking (smoker/non-smoker).
- Number of present teeth—the number of teeth present in the oral cavity at the time of clinical examination.
- Approximal Plaque Index (API)—an index assessing oral hygiene status based on the presence of dental plaque on approximal surfaces, expressed as a percentage.
- Bleeding on Probing (BOP)—an indicator of gingival bleeding upon probing, expressed as the percentage of sites showing bleeding.
- Probing Pocket Depth (PPD)—the depth of periodontal pockets measured in millimeters using a periodontal probe.
- Maximum Clinical Attachment Loss (CAL max)—the maximum value of clinical attachment loss recorded for a given patient, expressed in millimeters.

These clinical parameters were used as input variables in the periodontal disease classification process and as variables analyzed by the artificial intelligence models.

### 2.4. Expert Reference Classification

For each case, a reference diagnosis was established, which served as the benchmark for evaluating the classification accuracy of the artificial intelligence models.

The assessment was performed independently by two experienced periodontology specialists with extensive clinical experience. In cases of disagreement, a consensus discussion was conducted to establish the final reference diagnosis and to obtain Cohen’s kappa coefficient = 1, where available. Each case was classified according to the current periodontal disease classification, including:

- disease stage (Stage I–IV),
- disease grade (Grade A–C).

In cases of disagreement between the experts, a consensus discussion was conducted to determine the final reference classification.

A dental examination was conducted for all patients, including the assessment of the following parameters: the number of remaining teeth, approximal plaque index (API), bleeding on probing (BoP), probing depth (PD), and clinical attachment loss (CAL). Measurements were obtained using a UNC 15 periodontal probe with a cylindrical design, a 15 mm scale, a 1.75° taper, and a tip diameter of 0.5 mm [21]. Patients with periodontitis can be categorized into four stages, which reflect the severity, extent, and complexity of the disease. Additionally, three grades are used to evaluate the risk of disease progression as well as the potential impact on the patient’s systemic health [5].

### 2.5. Case Analysis Using a Large Language Model

The standardized clinical cases were subsequently analyzed using a large language model (LLM). The large language model used in this study was GPT-4o (OpenAI, San Francisco, CA, USA, 2024). All analyses were performed using identical prompts and standardized structured clinical input data. The deterministic settings (temperature = 0, when available) were applied to minimize output variability. The outputs were recorded directly without post-processing or manual correction before comparison with the expert reference classification.

For each patient, the model received a structured description of the available clinical and demographic variables derived from the dataset. The information provided to the model included:

- patient sex,
- patient age,
- smoking status,
- number of present teeth,
- Approximal Plaque Index (API),
- Bleeding on Probing (BOP),
- Probing Pocket Depth (PPD),
- maximum Clinical Attachment Loss (CAL max).

Each case was presented to the model together with the prompt:

“What is the stage and grade?”

Based on the provided clinical parameters, the model was asked to determine the stage and grade of periodontal disease according to the current periodontal disease classification. To ensure consistency in the evaluation process, all cases were analyzed using the same prompt structure and identical input format. The outputs generated by the model were then recorded and compared with the expert reference classification to assess the correctness of the predicted stage and grade.

### 2.6. Neural Network Model

Artificial Neural Network (ANN) models were developed using the neural network simulation software Statistica Automated Neural Networks (TIBCO Software Inc., Palo Alto, CA, USA, 2017; Statistica—data analysis software system, version 13; https://www.tibco.com, accessed on 1 September 2021). To achieve optimal performance, 100 multilayer perceptron (MLP) neural network models were generated. The models functioned as classification networks, whose task was to assign patients to appropriate diagnostic categories corresponding to both the stage of the disease (stage) and the rate of disease progression (grade) in periodontitis. The dataset was randomly divided into a training subset (*n* = 20) and a testing subset (*n* = 90). Neural network development was performed using the training subset, whereas performance evaluation was additionally examined on the complete dataset to enable comparison of classification patterns across all available cases. To reduce the risk of overfitting, model selection was based on automated optimization procedures implemented within the Statistica Automated Neural Networks framework.

During model development, supervised learning was applied, in which the model learns from previously labeled reference data. Network parameters, including the number of hidden neurons, the error function (sum of squares or cross-entropy), and the activation functions of hidden and output neurons (linear, logistic, hyperbolic tangent, exponential, or sine), were automatically selected by the program’s optimization algorithm.

### 2.7. Evaluation of Classification Accuracy

The accuracy of the models was evaluated by comparing the classifications generated by the artificial intelligence models with the reference diagnosis established by experts.For each model, the following metrics were calculated:

- classification accuracy,
- confusion matrices,
- Cohen’s kappa coefficient for the classification of disease stage and grade.

In addition, a qualitative analysis of diagnostic errors was performed in order to identify the most common types of misclassifications made by each model.

In our analysis, the “No periodontitis” category was not intended to represent an additional grade category. Rather, it was included in the confusion matrices to capture situations in which the AI model incorrectly identified a patient as having no periodontitis when the expert diagnosis indicated periodontitis, or vice versa. Therefore, the confusion matrices were designed to reflect the complete diagnostic output generated by the models, including errors related to disease presence or absence.

To avoid misunderstanding, we have revised Section 2 and the legends of the confusion matrices to clarify that “No periodontitis” represents a diagnostic category reflecting disease absence and is displayed only to illustrate model misclassification between healthy and diseased cases. It should not be interpreted as a periodontal grade within the 2017 classification system.

### 2.8. Analysis of Classification Errors

To better understand the limitations of the analyzed models, a detailed analysis of classification errors was performed. Errors were categorized into several types:

- stage underestimation,
- stage overestimation,
- grade misclassification,
- incorrect assessment of disease extent,
- failure to account for risk factors, such as smoking or diabetes,
- logical errors resulting from incorrect interpretation of input data.

For each error category, the number and percentage of cases in which the specific type of error occurred were calculated.

### 2.9. Statistical Analysis

Statistical analysis was performed using software (TIBCO Software Inc., Palo Alto, CA, USA, 2017; Statistica—data analysis software system, version 13; https://www.tibco.com, accessed on 1 September 2021). Continuous variables were presented as means and standard deviations, whereas categorical variables were expressed as counts and percentages.

Comparisons between groups, such as correctly and incorrectly classified cases, were performed using the Student’s *t*-test or Mann–Whitney U test for continuous variables, depending on data distribution, and the chi-square (χ^2^) test or Fisher’s exact test for categorical variables. The agreement between model predictions and expert reference classification was assessed using Cohen’s kappa coefficient (κ), with interpretation based on standard agreement categories. Diagnostic performance of the models was evaluated using accuracy (percentage of correct classifications) and confusion matrices for both stage and grade classification. Differences in performance between the large language model (LLM) and the neural network (NN) were analyzed using the McNemar test for paired nominal data. When expected cell counts were low or zero, the exact McNemar test based on the binomial distribution was applied. To identify factors associated with incorrect classification, logistic regression analysis was conducted. Univariate (univariable) logistic regression was used to assess the relationship between each independent variable and the outcome (incorrect classification), and variables with *p* < 0.1 were included in multivariable logistic regression models. The results were reported as odds ratios (ORs with 95% confidence intervals (95% CI)). Confidence intervals for accuracy were calculated using binomial methods, and 95% confidence intervals for Cohen’s kappa were estimated based on standard error calculations. Additionally, a detailed analysis of classification errors was performed, and differences in the frequency of specific error types between models were evaluated using the McNemar test. A *p*-value < 0.05 was considered statistically significant.

## 3. Results

### 3.1. Characteristics of the Study Population

The study population consisted of 110 patients aged between 24 and 61 years. The mean age of the participants was 45.7 (7.7) years. Among the included patients, 38 (35%) were male, 72 (65%) were female, and 23% were smokers. The mean number of present teeth was 25.4 (3.4). The mean periodontal parameters were API: 76.2 (23.9%), BOP: 67.3 (30.8%), PPD: 6.8 (2.9) mm, and maximum CAL: 5.3 (3.5) mm.

### 3.2. Expert Reference Classification

Based on the expert assessment, patients were classified according to the stage and grade of periodontitis. The distribution of disease stages showed that 10 patients (9%) were classified as no periodontitis, 12 patients (11%) were classified as Stage I, 19 patients (17%) as Stage II, 42 patients (38%) as Stage III, and 27 patients (25%) as Stage IV. Regarding the rate of disease progression, Grade A was identified in 15 patients (15%), Grade B in 43 patients (43%), and Grade C in 42 patients (42%).

### 3.3. Classification Accuracy of the Large Language Model

The classifications generated by the large language model (LLM) were compared with the expert reference classification. The large language model achieved an accuracy of 62% (95% CI: 53–71%) for stage classification and 63% (95% CI: 54–72%) for grade classification. The agreement between the large language model and the expert reference classification for stage assessment was κ = 0.48 (95% CI: 0.36–0.60), indicating a moderate level of agreement. The large language model demonstrated moderate agreement with expert classification for both stage and grade (κ = 0.48), with similar confidence intervals (95% CI: 0.36–0.60), indicating consistent but limited diagnostic performance. Detailed comparisons between the expert and model classifications are presented in the confusion matrices for stage and grade, shown in Table 1 and Table 2.

The confusion matrix comparing the stage classification generated by the large language model (LLM) with the expert reference classification is presented in Table 1. The model correctly classified all 10 patients without periodontitis, showing perfect agreement with the expert diagnosis in this category. However, the LLM demonstrated lower accuracy for Stage I, correctly identifying only 1 out of 12 cases, while 11 cases were incorrectly classified as no periodontitis. For Stage II, the model correctly classified 11 cases, while several misclassifications occurred, mainly into Stage I (3 cases) and Stage III (3 cases), with one case misclassified as Stage IV. In the Stage III group, the model correctly identified 33 patients, although 7 cases were overestimated as Stage IV and 2 cases were underestimated as Stage II. For Stage IV, the model correctly classified 13 cases, but 13 cases were underestimated as Stage III, and one case was misclassified as having no periodontitis. Overall, the most frequent misclassifications occurred between adjacent disease stages, particularly between Stage III and Stage IV, indicating that the model had difficulty distinguishing between more advanced stages of periodontitis.

The confusion matrix for grade classification comparing the predictions of the large language model (LLM) with the expert reference classification is presented in Table 2. The model correctly identified all 10 patients without periodontitis, showing complete agreement with the expert diagnosis in this category. However, the performance was lower for Grade A, where only 2 cases were correctly classified, while 11 cases were incorrectly classified as no periodontitis and 2 cases as Grade B. For Grade B, the LLM correctly classified 24 cases, while 15 cases were underestimated as Grade A, 2 cases were overestimated as Grade C, and 2 cases were misclassified as no periodontitis. In the Grade C group, the model correctly classified 33 patients, with 7 cases underestimated as Grade B and 2 cases as Grade A. Overall, the most common errors occurred between adjacent grades, particularly between Grade B and Grade C, suggesting that the model had difficulty distinguishing between moderate and rapidly progressing forms of periodontitis.

### 3.4. Classification Accuracy of the Neural Network

The neural network model was also used to classify patients according to the stage and grade of periodontitis. The neural network achieved an accuracy of 85% (95% CI: 78–92%) for stage classification and 79% (95% CI: 71–87%) for grade classification. The agreement between the neural network and the expert reference classification for stage assessment was κ = 0.79 (95% CI: 0.70–0.88), indicating a substantial level of agreement. The agreement between the neural network and the expert reference classification for grade assessment was κ = 0.67 (95% CI: 0.57–0.77), indicating a moderate to substantial level of agreement. These results suggest that the neural network demonstrated a relatively high consistency with expert assessments, particularly in the classification of disease stage. Detailed comparisons between the expert and Neural Network are presented in the confusion matrices for stage and grade, shown in Table 3 and Table 4.

The confusion matrix comparing stage classification generated by the neural network with the expert reference classification is presented in Table 3. The neural network correctly classified all 10 patients without periodontitis, showing complete agreement with the expert diagnosis in this category. For Stage I, the model correctly identified 8 cases, while 3 cases were overestimated as Stage II, and 1 case was underestimated as having no periodontitis. In the Stage II group, the neural network correctly classified 13 patients, whereas 6 cases were overestimated as Stage III. For Stage III, the model correctly classified 40 cases, with 2 cases misclassified as Stage IV. In the Stage IV category, 22 cases were correctly classified, while 4 cases were underestimated as Stage III, and 1 case was misclassified as no periodontitis. Overall, most misclassifications occurred between adjacent stages, particularly between Stage II and Stage III, as well as between Stage III and Stage IV, indicating that the neural network had difficulty distinguishing between neighboring stages of periodontitis severity.

The confusion matrix for grade classification generated by the neural network compared with the expert reference classification is presented in Table 4. The neural network correctly identified 6 out of 10 patients without periodontitis, while 3 cases were misclassified as Grade B and 1 case as Grade C. For Grade A, the model correctly classified 5 cases, whereas 9 cases were overestimated as Grade B and 1 case as Grade C. In the Grade B group, the neural network correctly identified 39 cases, while 4 cases were overestimated as Grade C. For Grade C, the model correctly classified 37 cases, with 5 cases underestimated as Grade B. Overall, most misclassifications occurred between adjacent grades, particularly between Grade B and Grade C, indicating that the neural network had some difficulty distinguishing between moderate and rapidly progressing forms of periodontitis.

### 3.5. Comparative Performance of the Models

The comparison of classification performance between the large language model (LLM) and the neural network (NN) is presented in Table 5. The large language model (LLM) achieved an accuracy of 62% (95% CI: 53–71%) for stage classification and 63% (95% CI: 54–72%) for grade classification. The agreement with the expert reference diagnosis was moderate, with Cohen’s kappa values of 0.48 (95% CI: 0.36–0.60) for both stage and grade. In contrast, the neural network (NN) demonstrated substantially higher performance, achieving 85% accuracy (95% CI: 78–92%) for stage classification and 79% (95% CI: 71–87%) for grade classification. The agreement with expert classification was substantial for stage (κ = 0.79; 95% CI: 0.70–0.88) and moderate to substantial for grade (κ = 0.67; 95% CI: 0.57–0.77). Overall, the neural network outperformed the large language model in both accuracy and agreement with expert diagnosis, particularly in the classification of disease stage.

The McNemar test showed a statistically significant difference in classification accuracy between the large language model and the neural network (*p* < 0.0001), indicating superior performance of the neural network model.

### 3.6. Analysis of Classification Errors

#### 3.6.1. Error Analysis in Stage and Grade Classification

The distribution of error types in stage classification for the large language model (LLM) and the neural network (NN) is presented in Table 6. The large language model demonstrated a significantly higher frequency of stage underestimation compared to the neural network (16.4% vs. 3.6%, *p* = 0.0005). Similarly, misclassification of periodontitis cases as no periodontitis was significantly more frequent in the LLM (11.8% vs. 1.8%, *p* = 0.0026). Errors between adjacent stages were also more common in the LLM (25.5%) than in the neural network (13.6%), with this difference reaching statistical significance (*p* = 0.0209). In contrast, stage overestimation occurred at the same frequency in both models (10.0% vs. 10.0%) and did not differ significantly (*p* = 0.7728). Distant stage errors (misclassification by two or more stages) were rare, occurring only in the LLM (2.7%) and not observed in the neural network; however, this difference was not statistically significant (*p* = 0.2500).

Overall, these findings indicate that the large language model was more prone to underestimating disease severity and misclassifying cases, whereas the neural network showed better performance and fewer clinically significant errors in stage classification.

The distribution of error types in grade classification for the large language model (LLM) and the neural network (NN) is presented in Table 7. The large language model demonstrated a significantly higher frequency of grade underestimation compared to the neural network (21.8% vs. 4.5%, *p* = 0.0002). In addition, misclassification of periodontitis cases as no periodontitis occurred exclusively in the LLM (11.8% vs. 0.0%, *p* = 0.0005), indicating a tendency of the model to underestimate disease presence. In contrast, the neural network showed a significantly higher rate of grade overestimation (16.4% vs. 3.6%, *p* = 0.0012), suggesting a tendency to assign more advanced grades of disease progression. Errors between adjacent grades were more frequent in the LLM (23.6%) than in the neural network (10.9%), with the difference being statistically significant (*p* = 0.0108). Distant grade errors were relatively rare in both models (1.8% for LLM vs. 4.5% for NN) and did not differ significantly (*p* = 0.4497).

Overall, these results indicate that the large language model was more prone to underestimating disease progression and misclassifying cases as healthy, whereas the neural network tended to overestimate disease severity, although it produced fewer errors in adjacent classifications.

#### 3.6.2. Factors Associated with Incorrect Stage and Grade Classification

The factors associated with incorrect stage classification by the large language model (LLM) are presented in Table 8. No statistically significant differences were observed between correctly and incorrectly classified cases for any of the analyzed variables. The mean age was similar in both groups (45.5 ± 8.5 vs. 45.9 ± 6.4 years, *p* = 0.7676), and there were no significant differences in sex distribution (38.2% vs. 28.6% males, *p* = 0.3004) or smoking status (23.5% vs. 21.4%, *p* = 0.7984). Similarly, clinical parameters did not differ significantly between the groups. The mean number of teeth (25.0 ± 3.5 vs. 25.9 ± 3.1, *p* = 0.2232), API (77.2 ± 23.9% vs. 74.7 ± 24.1%, *p* = 0.5203), BOP (67.6 ± 32.1% vs. 66.6 ± 29.0%, *p* = 0.8972), PPD (6.9 ± 2.9 mm vs. 6.6 ± 2.8 mm, *p* = 0.5404), and maximum CAL (5.2 ± 3.4 mm vs. 5.5 ± 3.8 mm, *p* = 0.7654) were comparable in correctly and incorrectly classified cases.

Overall, these results indicate that none of the analyzed demographic or clinical variables were significantly associated with incorrect stage classification by the large language model.

The factors associated with incorrect grade classification by the large language model (LLM) are presented in Table 9. A statistically significant association was observed only for smoking status, which was markedly more frequent in correctly classified cases compared to incorrectly classified cases (34.8% vs. 2.4%, *p* < 0.0001). This finding suggests that the model performed better in patients who were smokers. No significant differences were found for the remaining variables. The mean age was slightly higher in the incorrectly classified group (47.0 ± 7.0 vs. 44.9 ± 8.1 years, *p* = 0.1805), although this difference was not statistically significant. Similarly, sex distribution (37.7% vs. 29.3% males, *p* = 0.3696) and the number of teeth (25.0 ± 3.7 vs. 26.1 ± 2.8, *p* = 0.1066) did not differ significantly between the groups. Clinical periodontal parameters, including API (77.7 ± 23.6% vs. 73.9 ± 24.5%, *p* = 0.3717), BOP (68.2 ± 30.6% vs. 65.7 ± 31.6%, *p* = 0.6363), PPD (7.1 ± 3.0 mm vs. 6.3 ± 2.6 mm, *p* = 0.1533), and maximum CAL (5.5 ± 3.5 mm vs. 5.0 ± 3.6 mm, *p* = 0.4563) were also comparable between correctly and incorrectly classified cases.

Overall, these results indicate that smoking status was the only factor significantly associated with incorrect grade classification by the large language model, while other demographic and clinical variables showed no significant influence.

The factors associated with incorrect stage classification by the neural network (NN) are presented in Table 10. No statistically significant differences were observed between correctly and incorrectly classified cases for any of the analyzed variables. The mean age was comparable between groups (45.5 ± 7.6 vs. 46.8 ± 8.5 years, *p* = 0.5361), and no significant differences were found in sex distribution (35.5% vs. 29.4% males, *p* = 0.6283) or smoking status (21.5% vs. 29.4%, *p* = 0.4745). Similarly, no significant differences were identified for clinical parameters. The number of teeth (25.5 ± 3.1 vs. 24.8 ± 4.9, *p* = 0.9341), API (76.0 ± 23.9% vs. 77.9 ± 24.6%, *p* = 0.8817), BOP (66.8 ± 30.7% vs. 69.9 ± 32.4%, *p* = 0.6914), PPD (7.0 ± 2.9 mm vs. 5.8 ± 2.5 mm, *p* = 0.1181), and maximum CAL (5.4 ± 3.5 mm vs. 4.8 ± 3.5 mm, *p* = 0.4592) were similar in both groups.

Overall, these findings indicate that none of the analyzed demographic or clinical variables were significantly associated with incorrect stage classification by the neural network.

The factors associated with incorrect grade classification by the neural network (NN) are presented in Table 11. Several variables were significantly associated with incorrect classification. Patients in the incorrectly classified group had a higher number of teeth compared to correctly classified cases (26.8 ± 2.3 vs. 25.0 ± 3.5, *p* = 0.0132). In addition, incorrectly classified cases were characterized by lower PPD values (5.5 ± 2.4 mm vs. 7.2 ± 2.9 mm, *p* = 0.0061) and lower maximum CAL values (4.0 ± 3.9 mm vs. 5.7 ± 3.4 mm, *p* = 0.0414). No statistically significant differences were observed for age, although a trend toward lower age in incorrectly classified patients was noted (43.3 ± 7.2 vs. 46.3 ± 7.8 years, *p* = 0.0599). Similarly, sex distribution (37.9% vs. 21.7% males, *p* = 0.1464) and smoking status (24.1% vs. 17.4%, *p* = 0.4923) did not differ significantly between groups. Other clinical parameters, including API (77.1 ± 24.0% vs. 73.0 ± 23.7%, *p* = 0.3959) and BOP (69.4 ± 30.6% vs. 59.2 ± 31.2%, *p* = 0.1528), were also comparable between correctly and incorrectly classified cases.

Overall, these results indicate that incorrect grade classification by the neural network was associated with lower severity of periodontal parameters (PPD and CAL) and a higher number of remaining teeth, suggesting that the model had greater difficulty in accurately classifying less advanced or borderline cases of disease progression.

#### 3.6.3. Logistic Regression Analysis of Factors Associated with Incorrect Stage and Grade Classification

The results of the univariate logistic regression analysis of factors associated with incorrect stage classification are presented in Table 12. No statistically significant associations were found between the analyzed variables and the risk of incorrect stage classification for either the large language model (LLM) or the neural network (NN). The odds ratios (ORs) for all variables were close to 1, and the confidence intervals included the null value, indicating no significant effect. For the LLM, none of the demographic or clinical variables, including age, sex, smoking status, number of teeth, or periodontal parameters (API, BOP, PPD, CAL), were significantly associated with incorrect classification (all *p* > 0.05). Similarly, no significant associations were observed for the neural network. A non-significant trend toward lower odds of misclassification with increasing PPD values was noted (OR = 0.85; *p* = 0.1311), but this did not reach statistical significance.

Overall, these findings suggest that errors in stage classification were not significantly associated with the analyzed clinical or demographic variables, indicating that misclassification may be more related to model-specific limitations rather than input data characteristics.

The results of the univariate and multivariable logistic regression analysis of factors associated with incorrect grade classification are presented in Table 13. For the large language model (LLM), smoking status was the only variable significantly associated with incorrect classification. In the univariate analysis, smoking was associated with a lower risk of misclassification (OR = 0.05; 95% CI: 0.01–0.36; *p* = 0.0034), and this association remained significant in the multivariable model (adjusted OR = 0.05; *p* = 0.0034). No significant associations were observed for other variables, including age, sex, number of teeth, and clinical periodontal parameters (API, BOP, PPD, CAL). For the neural network (NN), the univariate analysis showed significant associations for the number of teeth (OR = 1.28; *p* = 0.0295), PPD (OR = 0.78; *p* = 0.0161), and CAL max (OR = 0.87; *p* = 0.0458). However, in the multivariable analysis, none of these variables remained statistically significant, although trends were observed for the number of teeth (adjusted OR = 1.22; *p* = 0.0773) and PPD (adjusted OR = 0.73; *p* = 0.1076).

Overall, these findings indicate that smoking was a key determinant of classification performance in the LLM, whereas in the neural network, clinical parameters appeared to influence misclassification in univariate analysis, but their effects diminished after adjustment for other variables.

Given the exploratory nature of the study and the relatively large number of statistical comparisons, *p*-values should be interpreted cautiously.

## 4. Discussion

The present study compared the diagnostic performance and classification error patterns of a large language model (LLM) and a neural network (NN) in the staging and grading of periodontitis according to the 2017 Classification of Periodontal and Peri-Implant Diseases and Conditions. The comparison between the neural network and the large language model should not be interpreted as a direct competition between equivalent AI systems, but rather as an exploratory evaluation of two fundamentally different AI paradigms applied to the same clinical classification task. The neural network was specifically developed using supervised learning on structured periodontal data, whereas the LLM represents a general-purpose model not specifically optimized for periodontal diagnostics. Therefore, the observed differences likely reflect distinctions in task specialization, optimization strategy, and training objectives.

This study evaluated the diagnostic accuracy of neural networks and the large language model when classifying periodontitis cases using the 2017 Classification of Periodontal and Peri-Implant Diseases and Conditions. The results indicate that neural networks tended to overestimate disease severity, while large language models tended to underestimate the severity of disease. The large language model achieved the highest accuracy in identifying patients without periodontitis, and most errors occurred while classifying patients with stage I, compared with stage IV of periodontitis. In grading, the LLM made most errors while estimating grade B. Misclassification predominantly occurred between adjacent disease stages, particularly between Stage III and Stage IV, indicating that the model had difficulty in distinguishing between more advanced stages of periodontitis and their extension. The neural network demonstrated substantially superior diagnostic performance in both stage and grade classification. Overall, most misclassifications made by the neural network occurred between adjacent stages, particularly between Stage II and Stage III, as well as between Stage III and Stage IV, indicating that the neural network had difficulty in distinguishing between neighboring stages of periodontitis severity and extension. In grading, most misclassifications occurred between adjacent grades, particularly between Grade B and Grade C. The accuracy of LLM in stage and grade evaluation was 62% and 63%, respectively, and for the neural network was 85% and 79%, respectively. Overall, the neural network outperformed the large language model in both accuracy and agreement with expert diagnosis, particularly in the classification of disease stage. The tendency of the LLM to underestimate disease severity may reflect limitations when interpreting periodontal parameters without domain-specific optimization. In contrast, the neural network demonstrated higher sensitivity to advanced disease patterns but occasionally overestimated progression severity, possibly reflecting bias toward more severe classifications within the training data. The clinical implications of AI-related diagnostic errors should be carefully considered. Underestimation of disease severity may delay periodontal intervention and lead to progression of tissue destruction, whereas overestimation may contribute to unnecessary treatment intensity and overtreatment. Therefore, evaluation of AI systems should include not only overall accuracy but also the direction, severity, and potential clinical consequences of diagnostic errors.

One of the most interesting findings of the present study was the significant association between smoking status and improved grade classification performance of the LLM. Smoking was associated with markedly lower odds of incorrect classification (OR = 0.05), and this association remained significant after multivariable adjustment. Several explanations may account for this observation. First, smoking represents one of the most explicit and heavily weighted risk modifiers within the 2017 periodontitis grading system. Because grading incorporates smoking directly as a determinant of progression risk, the presence of smoking may provide the LLM with a strong and unambiguous contextual cue facilitating assignment toward higher disease grades, particularly Grade C. In contrast, non-smoker cases may require more nuanced interpretation of multiple interacting periodontal parameters, increasing diagnostic ambiguity.

Accurate periodontal staging and grading are clinically important not only for diagnosis itself but also for treatment planning and long-term disease management. Contemporary periodontal therapy increasingly incorporates individualized and minimally invasive approaches, including adjunctive antimicrobial and bioactive therapies. Recent studies by Butera et al. demonstrated the potential benefits of novel adjunctive approaches in non-surgical periodontal therapy, including antimicrobial gels containing postbiotics, lactoferrin, and Aloe barbadensis leaf juice powder, as well as ozonized gels as alternatives to conventional chlorhexidine-based protocols. These studies highlight the growing importance of personalized periodontal management strategies and reinforce the need for accurate and reproducible diagnostic classification systems that may support appropriate therapeutic decision-making. In this context, artificial intelligence systems capable of assisting clinicians in periodontal classification may potentially contribute to improved standardization and optimization of periodontal care.

The integration of artificial intelligence into periodontology should therefore be viewed not only from the perspective of diagnostic automation but also as a potential supportive component of precision dentistry workflows. Reliable AI-assisted classification may facilitate earlier identification of disease severity, improve consistency in treatment planning, and support selection of individualized therapeutic strategies in daily clinical practice [22,23].

Recent evidence in dental artificial intelligence research further supports the importance of evaluating AI systems beyond simple accuracy metrics. Tayeb et al. demonstrated that large language models, including ChatGPT-5 (OpenAI, San Francisco, CA, USA), Gemini-Pro (Google DeepMind, London, UK), and DeepSeek (DeepSeek, Hangzhou, China), can achieve performance comparable to clinicians in identifying clinically relevant drug–drug interactions in dental therapy; however, their performance varied depending on the complexity and clinical framing of the task, highlighting the importance of context-dependent reliability rather than overall accuracy alone. Similarly, a randomized controlled trial by Pul et al. investigated the impact of AI-assisted diagnosis of periapical radiolucencies and showed that AI support can significantly influence diagnostic decision-making, particularly by improving consistency and reducing variability among clinicians. Importantly, this study emphasized that the clinical value of AI systems lies not only in their standalone diagnostic performance but also in their role as decision-support tools that may modify human diagnostic behavior. Together, these findings align with the present study by reinforcing that AI evaluation in dentistry should incorporate error structure, clinical context, and potential effects on decision-making processes, rather than relying exclusively on aggregate performance measures [24,25].

The 2017 classification system was implemented to deal with imperfections of the classification from 1999 and mostly to emphasize the clinical approach [26]. While it provides a more organized and detailed diagnostic strategy that considers stage, grade, and extent, it has also brought additional difficulties for professionals. The uptake of this new system has varied, especially among general dentists, hygienists, and dental students, who view it as complicated, demanding, and unfamiliar. Studies have pointed out these challenges in implementing the system. New technologies like LLM or neural networks can assist general dentists, hygienists, or even students in their periodontitis diagnosis [27,28].

Most of the studies in periodontology that use artificial intelligence, like neural networks or large language models, take into consideration dental radiographs [29,30,31]. On the contrary, in our study, we compared a neural network with the large language model using clinical records of patients examined independently by two experienced periodontists. Due to the limited number of studies in the literature, a comparison between the findings from other studies and the findings of our study would be challenging. Ji E. et al. in their study assessed the diagnostic precision of five dental professionals and ChatGPT-4 when classifying periodontitis cases using the 2017 Classification of Periodontal and Peri-Implant Diseases and Conditions. Their findings show that although ChatGPT-4 exhibited a reasonable level of diagnostic ability, it did not outperform most individual clinicians. The accuracy of ChatGPT-4 in stage and grade evaluation was 70% and 60%, respectively [32]. Tastan Eroglu Z. et al. in their study evaluated the accuracy of ChatGPT-3,5 in periodontitis classification when clinical records were measured by periodontists. Their results show that ChatGPT-3,5 successfully identified the periodontitis stage and grade in 59.5% and 50.5% of cases [33]. In Ertaş K. et al.’s study, the classification accuracy was evaluated with the usage of seven supervised machine learning algorithms, using clinical attributes. The most precise turned out to be the tree algorithm (97.2%) and the random forest model (96.5%). Moreover, they concluded that even four instead of ten clinical attributes are sufficient for a model to make a good diagnosis [34]. In Nantakeeratipat et al. GPT-4o and Gemini Pro were evaluated in periodontitis staging and grading assessment. While GPT-4o’s staging accuracy was 70.5%, its grading accuracy was up to 100%, indicating that its errors were just limited to staging. Gemini Pro, however, struggled with both, achieving only 36.2% accuracy on staging and 78.3% on grading [35]. Lafourcade et al. examined the accuracy of four different LLMs in comparison with expert-provided answers in the field of restorative dentistry and endodontics. The accuracy of models was up to 63%, overall, the models achieved promising levels of contextual understanding, suggesting relevance in this particular area, but authors also emphasize that the models should be used with caution, and more investigation in this area is needed. The large language models present several limitations in medical applications, including susceptibility to hallucinations, dependence on prompt formulation, limited explainability, and variability of generated outputs. These factors may reduce reliability and reproducibility in clinical decision-making and currently limit autonomous clinical implementation [36].

## 5. Conclusions

Within the limitations of this exploratory study, the task-specific neural network demonstrated higher diagnostic accuracy and stronger agreement with expert classification than the evaluated general-purpose large language model for periodontal staging and grading in this dataset. However, these findings should be interpreted in the context of the fundamentally different architectures, training paradigms, and intended applications of the evaluated artificial intelligence systems. The neural network was specifically developed and trained using supervised learning on structured periodontal data, whereas the large language model represents a general-purpose system not explicitly optimized for periodontal diagnosis.

Accordingly, the observed differences likely reflect differences in task specialization rather than direct superiority of one artificial intelligence approach over the other. Neural networks designed for domain-specific classification tasks may currently offer greater reliability in structured clinical decision-making, while large language models may provide broader accessibility and flexibility as supportive tools in clinical education and decision support.

Importantly, neither system should be considered a replacement for specialist clinical judgment. Both models should be viewed as adjunctive tools that may assist clinicians in interpreting complex periodontal data and supporting decision-making processes, rather than autonomous diagnostic systems.

Future advances in model architecture, domain-specific training, and integration of multimodal clinical data may further improve the performance and clinical utility of both approaches. Prospective multicenter validation studies are required to better define their role in routine periodontal practice and to ensure safe and effective implementation in clinical settings.

## 6. Limitations

The relatively small sample size and single-center retrospective design may limit the generalizability of the findings. In addition, external validation was not performed, and the possibility of model overfitting cannot be completely excluded.

An additional limitation is that the neural network performance metrics were calculated using classifications generated for the entire study dataset, including cases used during model development. Consequently, the reported results may overestimate the true generalization performance of the model and should be interpreted as exploratory.

Future multicenter studies using larger and more diverse datasets are required to confirm the reproducibility and clinical applicability of these findings. Unlike task-specific neural networks trained on periodontal datasets, the evaluated LLM was not specifically optimized for periodontal diagnostics, which may have influenced classification performance. In contrast to neural networks designed for specific tasks that have been trained using labeled dental datasets, LLM serves as a versatile language model that has undergone training on an extensive collection of public data. This extensive background might affect its accuracy in making diagnoses when compared to models focused on specialties. However, its availability and user-friendly language capabilities present opportunities for usefulness in both educational and healthcare environments as an additional resource [32,37].

A further important limitation of this study relates to the completeness of clinical input data used for periodontal classification. Although the models were instructed to classify periodontitis according to the 2017 Classification of Periodontal and Peri-Implant Diseases and Conditions [1,5], the available input variables were limited to age, sex, smoking status, number of teeth, API, BOP, PPD, and maximum CAL. Consequently, several key elements of the full classification system may not have been explicitly incorporated into the model inputs.

In particular, radiographic bone loss, tooth loss due to periodontitis, distribution and extent of disease, complexity factors, diabetes status, and indicators of progression were either unavailable or not directly included in the dataset. These variables are important components of the 2017 staging and grading framework and contribute substantially to accurate classification, especially in differentiating between stages and grades with similar clinical profiles.

Therefore, the classification process in this study should be interpreted as a simplified operational approximation of the 2017 system rather than a fully comprehensive implementation of all diagnostic criteria. This limitation may have influenced model performance and error patterns, particularly in more complex or borderline cases. Future studies should aim to incorporate complete multidimensional clinical, radiographic, and systemic datasets to ensure full alignment with the 2017 classification system and to improve the external validity of AI-based periodontal diagnostic models.

## Figures and Tables

**Table 1:** Confusion matrix for stage classification (Large Language Model vs. Expert).

Expert\LLM	No Periodontitis	Stage I	Stage II	Stage III	Stage IV
No periodontitis	10	0	0	0	0
Stage I	11	1	0	0	0
Stage II	1	3	11	3	1
Stage III	0	0	2	33	7
Stage IV	1	0	0	13	13

**Table 2:** Confusion matrix for grade classification (Large Language Model vs. Expert).

Expert\LLM	No Periodontitis	Grade A	Grade B	Grade C
No periodontitis	10	0	0	0
Grade A	11	2	2	0
Grade B	2	15	24	2
Grade C	0	2	7	33

**Table 3:** Confusion matrix for stage classification (Neural Network vs. Expert).

Expert\LLM	No Periodontitis	Stage I	Stage II	Stage III	Stage IV
No periodontitis	10	0	0	0	0
Stage I	1	8	3	0	0
Stage II	0	0	13	6	0
Stage III	0	0	0	40	2
Stage IV	1	0	0	4	22

**Table 4:** Confusion matrix for grade classification (Neural Network vs. Expert).

Expert\LLM	No Periodontitis	Grade A	Grade B	Grade C
No periodontitis	6	0	3	1
Grade A	0	5	9	1
Grade B	0	0	39	4
Grade C	0	0	5	37

**Table 5:** Comparison of classification performance between the Large Language Model and the Neural Network.

Model	Stage Accuracy (%)	Grade Accuracy (%)	Cohen’s Kappa (95%CI) (Stage)	Cohen’s Kappa (95%CI) (Grade)
LLM ^1^	62% (53–71%)	63% (54–72%)	0.48 (0.36–0.60)	0.48 (0.36–0.60)
NN ^2^	85% (78–92%)	79% (71–87%)	0.79 (0.70–0.88)	0.67 (0.57–0.77)

^1^ Large Language Model, ^2^ Neural Network.

**Table 6:** Types of errors in stage classification for the Large Language Model and the Neural Network.

Error Type	Definition	LLM ^1^ *n* (%)	NN ^2^ *n* (%)	*p*-Value
Stage underestimation	Model assigned a lower stage than expert	18 (16.4%)	4 (3.6%)	0.0005
Stage overestimation	Model assigned a higher stage than expert	11 (10.0%)	11 (10.0%)	0.7728
Misclassification to no periodontitis	Periodontitis classified as healthy	13 (11.8%)	2 (1.8%)	0.0026
Adjacent stage error	Misclassification between neighboring stages	28 (25.5%)	15 (13.6%)	0.0209
Distant stage error	Misclassification ≥ 2 stages away	3 (2.7%)	0 (0.0%)	0.2500

^1^ Large Language Model, ^2^ Neural Network.

**Table 7:** Types of errors in grade classification for the Large Language Model and the Neural Network.

Error Type	Definition	LLM ^1^ *n* (%)	NN ^2^ *n* (%)	*p*-Value
Grade underestimation	Model assigned a lower grade than expert	24 (21.8%)	5 (4.5%)	0.0002
Grade overestimation	Model assigned a higher grade than expert	4 (3.6%)	18 (16.4%)	0.0012
Misclassification of no periodontitis	Periodontitis classified as healthy	13 (11.8%)	0 (0.0%)	0.0005
Adjacent grade error	Misclassification between neighboring grades	26 (23.6%)	12 (10.9%)	0.0108
Distant grade error	Misclassification ≥ 2 grades away	2 (1.8%)	5 (4.5%)	0.4497

^1^ Large Language Model, ^2^ Neural Network.

**Table 8:** Factors associated with incorrect stage classification by the Large Language Model.

Variable	Correct Classification (*n* = 68)	Incorrect Classification (*n* = 42)	*p*-Value
Age (years), mean ± SD ^1^	45.5 ± 8.5	45.9 ± 6.4	0.7676
Sex (male), *n* (%)	26 (38.2%)	12 (28.6%)	0.3004
Smoking, *n* (%)	16 (23.5%)	9 (21.4%)	0.7984
Number of teeth, mean ± SD	25.0 ± 3.5	25.9 ± 3.1	0.2232
API (%), mean ± SD	77.2 ± 23.9	74.7 ± 24.1	0.5203
BOP (%), mean ± SD	67.6 ± 32.1	66.6 ± 29.0	0.8972
PPD (mm), mean ± SD	6.9 ± 2.9	6.6 ± 2.8	0.5404
CAL max (mm), mean ± SD	5.2 ± 3.4	5.5 ± 3.8	0.7654

^1^ Standard Deviation.

**Table 9:** Factors associated with incorrect grade classification by the large language model.

Variable	Correct Classification (*n* = 69)	Incorrect Classification (*n* = 41)	*p*-Value
Age (years), mean ± SD ^1^	44.9 ± 8.1	47.0 ± 7.0	0.1805
Sex (male), *n* (%)	26 (37.7%)	12 (29.3%)	0.3696
Smoking, *n* (%)	24 (34.8%)	1 (2.4%)	<0.0001
Number of teeth, mean ± SD	25.0 ± 3.7	26.1 ± 2.8	0.1066
API (%), mean ± SD	77.7 ± 23.6	73.9 ± 24.5	0.3717
BOP (%), mean ± SD	68.2 ± 30.6	65.7 ± 31.6	0.6363
PPD (mm), mean ± SD	7.1 ± 3.0	6.3 ± 2.6	0.1533
CAL max (mm), mean ± SD	5.5 ± 3.5	5.0 ± 3.6	0.4563

^1^ Standard Deviation.

**Table 10:** Factors associated with incorrect stage classification by the neural network.

Variable	Correct Classification (*n* = 93)	Incorrect Classification (*n* = 17)	*p*-Value
Age (years), mean ± SD ^1^	45.5 ± 7.6	46.8 ± 8.5	0.5361
Sex (male), *n* (%)	33 (35.5%)	5 (29.4%)	0.6283
Smoking, *n* (%)	20 (21.5%)	5 (29.4%)	0.4745
Number of teeth, mean ± SD	25.5 ± 3.1	24.8 ± 4.9	0.9341
API (%), mean ± SD	76.0 ± 23.9	77.9 ± 24.6	0.8817
BOP (%), mean ± SD	66.8 ± 30.7	69.9 ± 32.4	0.6914
PPD (mm), mean ± SD	7.0 ± 2.9	5.8 ± 2.5	0.1181
CAL max (mm), mean ± SD	5.4 ± 3.5	4.8 ± 3.5	0.4592

^1^ Standard Deviation.

**Table 11:** Factors associated with incorrect grade classification by the Neural Network.

Variable	Correct Classification (*n* = 87)	Incorrect Classification (*n* = 23)	*p*-Value
Age (years), mean ± SD ^1^	46.3 ± 7.8	43.3 ± 7.2	0.0599
Sex (male), *n* (%)	33 (37.9%)	5 (21.7%)	0.1464
Smoking, *n* (%)	21 (24.1%)	4 (17.4%)	0.4923
Number of teeth, mean ± SD	25.0 ± 3.5	26.8 ± 2.3	0.0132
API (%), mean ± SD	77.1 ± 24.0	73.0 ± 23.7	0.3959
BOP (%), mean ± SD	69.4 ± 30.6	59.2 ± 31.2	0.1528
PPD (mm), mean ± SD	7.2 ± 2.9	5.5 ± 2.4	0.0061
CAL max (mm), mean ± SD	5.7 ± 3.4	4.0 ± 3.9	0.0414

^1^ Standard Deviation.

**Table 12:** Univariate Logistic Regression Analysis of Factors Associated with Incorrect Stage Classification.

Variable	LLM ^1^ OR (95% CI)	*p*-Value	NN ^2^ OR ^3^ (95% CI)	*p*-Value
Age (years)	1.00 (0.96–1.06)	0.7796	1.02 (0.95–1.10)	0.5325
Sex (male)	0.65 (0.28–1.48)	0.3019	0.76 (0.25–2.34)	0.6290
Smoking	0.89 (0.35–2.24)	0.7984	1.52 (0.48–4.83)	0.4767
Number of teeth	1.09 (0.96–1.23)	0.1877	0.94 (0.82–1.09)	0.4155
API (%)	1.00 (0.98–1.01)	0.5982	1.00 (0.98–1.03)	0.7616
BOP (%)	1.00 (0.99–1.01)	0.8627	1.00 (0.99–1.02)	0.7023
PPD (mm)	0.96 (0.84–1.10)	0.5418	0.85 (0.69–1.05)	0.1311
CAL max (mm)	1.02 (0.91–1.14)	0.7268	0.95 (0.82–1.10)	0.4739

^1^ Large Language Model, ^2^ Neural Network, ^3^ Odds Ratio.

**Table 13:** Univariate and Multivariable Logistic Regression Analysis of Factors Associated with Incorrect Grade Classification.

Variable	LLM ^1^ OR (95% CI)	*p*-Value	LLM Adjusted OR (95% CI)	*p*-Value	NN ^2^ OR ^3^ (95% CI)	*p*-Value	NN Adjusted OR (95% CI)	*p*-Value
Age (years)	1.04 (0.98–1.09)	0.1808			0.95 (0.89–1.01)	0.0939		
Sex (male)	0.68 (0.30–1.57)	0.3707			0.46 (0.15–1.34)	0.1530		
Smoking	0.05 (0.01–0.36)	0.0034	0.05 (0.01–0.36)	0.0034	0.66 (0.20–2.16)	0.4945		
Number of teeth	1.12 (0.98–1.28)	0.0936			1.28 (1.03–1.60)	0.0295	1.22 (0.98–1.52)	0.0773
API (%)	0.99 (0.98–1.01)	0.4168			0.99 (0.97–1.02)	0.4575		
BOP (%)	1.00 (0.99–1.01)	0.6796			0.99 (0.97–1.00)	0.1637		
PPD (mm)	0.90 (0.78–1.03)	0.1337			0.78 (0.64–0.96)	0.0161	0.73 (0.50–1.07)	0.1076
CAL max (mm)	0.96 (0.86–1.07)	0.4862			0.87 (0.75–1.00)	0.0458	1.11 (0.85–1.44)	0.4619

^1^ Large Language Model, ^2^ Neural Network, ^3^ Odds Ratio.

## Data Availability

All data generated or analyzed during this study are included in this published article.
